# R Dump Converter without DC Link Capacitor for an 8/6 SRM: Experimental Investigation

**DOI:** 10.1155/2015/393629

**Published:** 2015-01-06

**Authors:** Pasumalaithevan Kavitha, Bhaskaran Umamaheswari

**Affiliations:** ^1^Department of Electrical and Electronics Engineering, Anna University, Chennai, Tamil Nadu 600025, India; ^2^Department of Electronics and Instrumentation Engineering, RMK Engineering College, Kavaraipettai, Tamil Nadu 601206, India

## Abstract

The objective of this paper is to investigate the performance of 8/6 switched reluctance motor (SRM) when excited with sinusoidal voltage. The conventional R dump converter provides DC excitation with the help of capacitor. In this paper the converter used is the modified R dump converter without DC link capacitor providing AC or sinusoidal excitation. Torque ripple and speed ripple are investigated based on hysteresis current control. Constant and sinusoidal current references are considered for comparison in both DC and AC excitation. Extensive theoretical and experimental investigations are made to bring out the merits and demerits of AC versus DC excitation. It is shown that the constructionally simple SRM can be favorably controlled with simple R dump converter with direct AC excitation without need for DC link capacitor. A 4-phase 8/6 0.5 kW SRM is used for experimentation.

## 1. Introduction

The switched reluctance motor (SRM) is the simplest and most efficient variable speed drive. It has high reliability and operates at very high speed. Generally SRM drives are excited with DC supply. AC voltage is rectified and smoothened by the DC link capacitor for DC excitation [1, 2]. The cost and performance of SRM drives are dependent on converter topologies and motor structure. There have been many converter topologies found in literature for the past two decades in order to meet the objectives such as faster excitation, faster demagnetization, high efficiency, and minimizing torque ripple and to draw minimum reactive power [3–8].

The single switch per phase converter like R dump, C dump [9], bifilar, and split DC link is cost effective [10]. In C dump the energy is stored in the capacitor; hence the efficiency is high. But the need for bulky capacitor and inductor makes the converter cost increase and losses are also increased. The conventional asymmetric H bridge converter is also used for independent control of voltage and current but it has disadvantages like high cost, necessity for isolated gate drives, and a requirement for complex control system. The SRM is capable of providing unidirectional torque for bidirectional current; hence it can be excited with matrix converters [11–14]. Sinusoidal excitation with overlapped phase currents is shown to reduce torque ripple and iron losses [15–17]. Single sided matrix converter with unidirectional current is shown to have the advantage of self-commutating in SRM [18]. Torque ripple can be minimized initially at the design stage itself and further by suitable control techniques. An inner current loop is introduced to have control over the torque. Hysteresis current control is the simple method but has the disadvantage of variable switching frequency, high current ripple, and consequent audible noise. PI/PID based fixed switching frequency control provides easier digital implementation with low current ripples and low audible noise, which show poor performance with varying operating conditions. Fuzzy logic, neural network, and adaptive and genetic algorithm based tuning of current shapes is shown to achieve fewer vibrations [19–23].

The objective of this paper is to provide simple converter and control strategies for SRM to be operated with direct AC excitation with minimum number of switching devices and storage elements. SRM can be operated with bidirectional currents with direct AC excitation. This requires bidirectional switches increasing the complexity of the converter. Hence the configuration used in this paper for excitation is a full bridge diode rectifier followed by modified R dump converter without any capacitor at both the DC link and R dump circuit. This facilitates unidirectional sinusoidal excitation. Hysteresis current control is employed with DC and sinusoidal current references. Performance of the machine is analysed in terms of torque ripple, speed ripple, and total harmonic distortion (THD) at the input. Theoretical and experimental investigations are made and the relative merits and demerits of sinusoidal versus DC excitation are brought out.

This paper is organized as follows.  Section 2 explains the conventional and modified R dump converter topology.  Section 3 provides dynamic modeling of the proposed drive system. Performance analysis of the SRM under DC and AC current references is discussed in Section 4. Experimental setup is discussed in Section 5 to show the effectiveness of the proposed converter.  Section 6 concludes this paper.

## 2. Converter Topology

**Figure 1:**
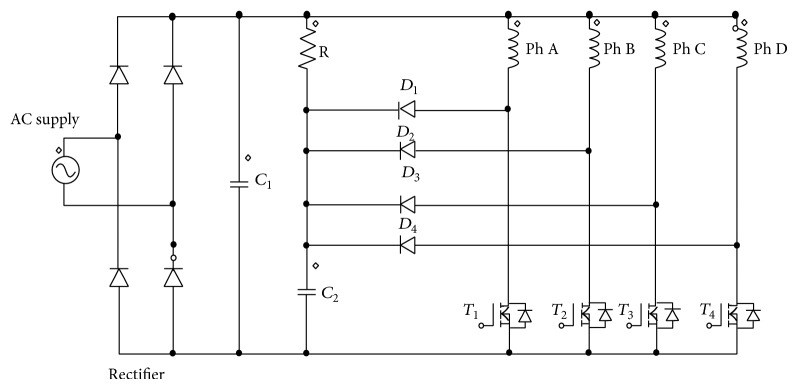
Conventional R dump converter for DC excitation.

**Figure 2:**
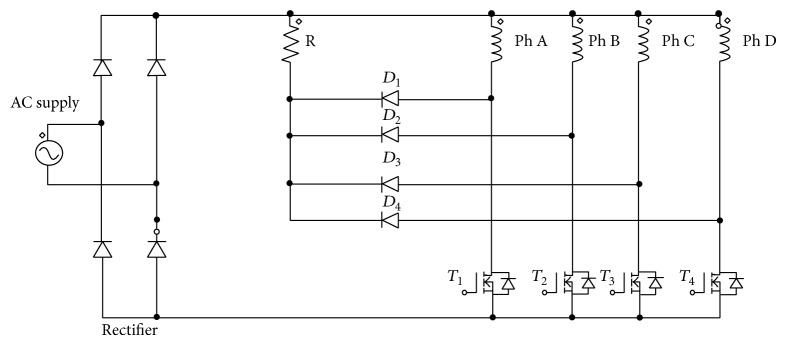
Modified R dump converter for AC or sinusoidal excitation.

The conventional and modified R dump converters are shown in Figures 1 and 2. The only difference is the absence of two capacitors *C*_1_ and *C*_2_. Capacitor *C*_1_ provides filtered DC to the conventional controller and capacitor *C*_2_ helps in utilizing the regenerative power from the phases. The converter has one transistor and one diode per phase of the SRM drive. The converter is fed from an AC supply with full bridge rectifier along with a DC link capacitor. When switches are turned on, the phase windings are excited. When switch (*T*_1_) is turned off, the current freewheels through *D*_1_, charging the DC link capacitor (*C*_1_) as well as the dump capacitor (*C*_2_), and later flows through the external resistor R. This resistor partially dissipates the energy stored in phase A. However, it has the disadvantage that, the current in phases will take longer time to extinguish when compared to recharging the source. The energy, in addition, is dissipated in a resistor; hence overall efficiency of the motor drive reduced.

The performance of SRM is investigated with a full bridge rectifier and a utility AC supply which is shown in Figure 2. The bulkier DC link capacitor is removed. The utility sinusoidal supply is rectified and applied to the converter. The converter is now compact and weighs less.

## 3. Dynamic Model of the Proposed Drive Topology 

**Figure 3:**
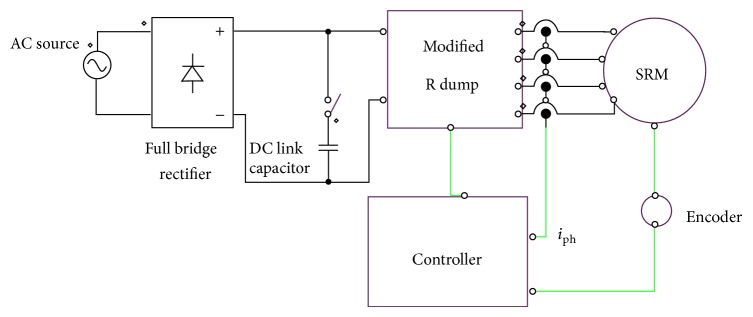
Block diagram of SRM drive.

**Figure 4:**
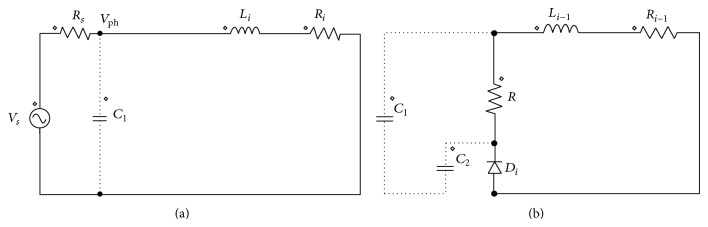
(a) Equivalent circuit of the incoming phase. (b) Equivalent circuit of the outgoing phase.

The functional block diagram of the proposer SRM drive is shown in Figure 3. At any given instant of time one of the phases will be excited based on the *θ*_ON_ and *θ*_OFF_ specifications. The excited switching device of the preceding phase will be put to off condition. This leads to the situation where both the incoming and outgoing phases will carry current. The dynamic model of the drive can be derived based on the equivalent circuits shown in Figures 4(a) and 4(b).

Figures 4(a) and 4(b) show the incoming and outgoing phases with modified converter. Dotted line represents the conventional R dump model. The dynamic model can be presented as given in the following equations. Let *i*_*i*_ be the current of the *i*th phase which is incoming and let *i*_*i*−1_ be the current of the *i* − 1th phase which is outgoing. Then the corresponding voltage equations are given by

(1) Vph=Rsii+Riii+dλiθi,iidt for    θ  €  θON,θOFF,0=Ri−1ii−1+dλi−1θi−1,ii−1dt+Rii−1for    θ  €  θON,θOFF,

where *V*_ph_ is the phase voltage, *R* is the resistance of the modified R dump converter, *R*_*i*_ and *R*_*i*−1_ represent the resistances of the respective phases, and *R*_*s*_ is the source resistance. Let *λ*_*i*_ and *λ*_*i*−1_ represent the flux linkages of the two phases as given by

(2) λi=Lmi,i−1(θi−1,ii−1)ii−1+Lii(θi,ii)iiλi−1=Lii−1θi−1,ii−1ii−1+Lmiθi,iiii,

where *L*_*ii*_ represents the self-inductance of the phases and *L*_*mi*,*i*−1_ represents the mutual inductance between the phases. Since SRM are characterized by concentrated winding, the mutual inductance is negligible. The developed torque equation and torque balance equations are given by

(3) $$\begin{array}{c} T_{d}=\frac{1}{2}\left[i_{i-1}^{2}\frac{\partial L_{i-1}}{\partial \theta_{i-1}}+i_{i}^{2}\frac{\partial L_{i}}{\partial \theta_{i}}\right],\\ T_{d}-T_{L}-J\frac{d\omega }{dt}-B\omega =0, \end{array}$$

where *T*_*d*_ is the developed torque, *T*_*L*_ is the load torque, *ω* is the angular speed, *J* is the moment of inertia, and *B* is the frictional coefficient. The self-inductance of the machine phases is assumed to take the cosine form which is considered along with saturation as defined by

(4) $$\begin{array}{c} L_{i}=\left(L_{0}+L_{1}\cos N_{r}\left(\theta_{i}+\theta_{ppi}\right)\right)e^{-ki_{i}}, \end{array}$$

where *L*_0_ and *L*_1_ are functions of average and difference of aligned and unaligned inductances, respectively, *N*_*r*_ is the number of rotor poles, *θ*_*pp*_ is the phase shift between the phases, and *k* is a constant chosen to represent saturation. Additional equations to be described in the presence of capacitors of the R dump are given by

(5) $$\begin{array}{c} C_{1}\frac{dV_{C1}}{dt}=I_{s}-I_{i}+I_{i-1},\\ C_{2}\frac{dV_{C2}}{dt}=I_{i-1}. \end{array}$$

Let the supply voltage be defined as follows:

(6) $$\begin{array}{c} V_{s}=V_{m}\text{mag}\left(\sin\omega t\right), \end{array}$$

where mag(·) means the magnitude of the variable. Using equations (1)–(6) the switching model of the SRM is simulated for the converters with and without DC link capacitor.

## 4. Performance Analysis

The simplest control for SRM can be the fixing of *θ*_ON_ and *θ*_OFF_ at constant value with hysteresis current control. The fixing of *θ*_ON_ and *θ*_OFF_ constant can be easily implemented with the help of optical sensors used for switching the phases. This will ensure continuous torque development from the phases consecutively one after the other. Hysteresis current control can be easily implemented with the help of hysteresis comparator.

The specification of the machine and the drive is given in Table 1. Control part is implemented through hardware in loop. Commutation pulses are generated based on the information from position sensor which is acquired through a National Instrumentations Data Acquisition (NI DAQ) card PCI 6251. Components of the modified R dump converter with necessary isolation and gate drivers used to commutate the phase current are given in Table 1. Hall effect current and voltage sensors are used for measuring the active phase voltage and current, respectively. The experimental setup is discussed in Section 5 based on which the performance analysis is done.

Performance of the SRM is analysed both theoretically and experimentally for two cases, namely, Case (i) DC and AC excitation with constant current reference and Case (ii) DC and AC excitation with sinusoidal current reference. The DC excitation considered in this paper is equivalent to the use of conventional R dump converter shown in Figure 1. The AC excitation referred to is equivalent to the use of modified R dump converter as shown in Figure 2. The rotor position is sensed with two optical sensors providing pulses for every 15-degree mechanical angle which is equal to the shift between the phases. The pulses obtained from the position sensors are used as *θ*_ON_ and *θ*_OFF_ which can be chosen as 0, 15, 30, 45, and 60 degrees. Detailed results are presented in the following sections.

**Figure 5:**
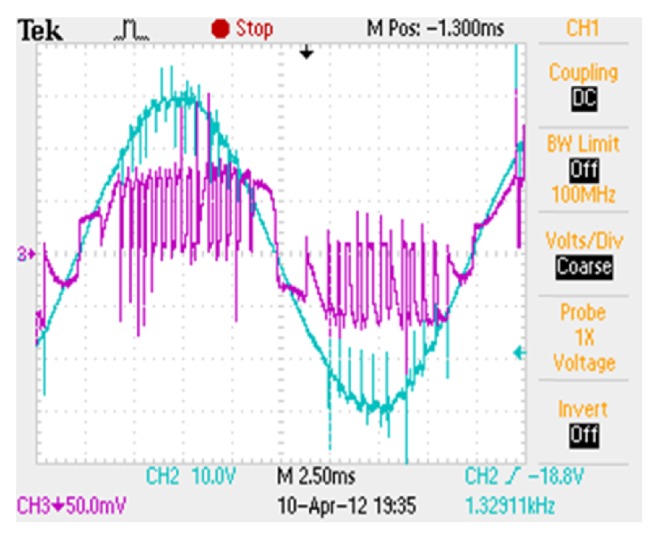
Supply voltage and current of modified converter with constant current reference.

*Case (i) DC and AC Excitation with Constant Current Reference*. The AC supply is fixed with peak to peak of 60 V. The supply voltage and current waveforms for the modified converter configuration are shown in Figure 5 for a constant current reference of 0.5 A. *θ*_ON_ is kept at 45° and *θ*_OFF_ is kept at 75° with dwell angle of 30°. The supply current is highly discontinuous in the presence of capacitor when compared to without capacitor configuration. It is worth analysing the power factor and total harmonic distortion for various values of current references.

**Figure 6:**
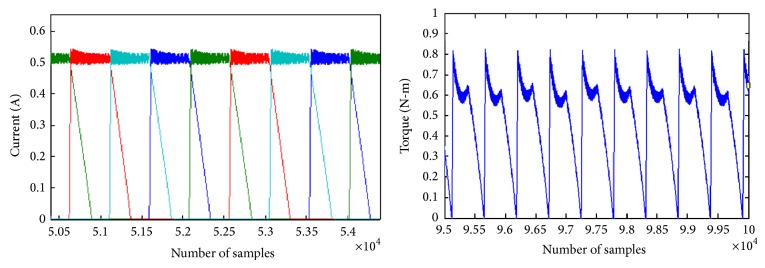
Phase currents and developed torque of SRM with 60 V DC excitation and constant current reference of 0.5 A.

The phase currents and developed torque waveforms for the DC excitation are given in Figure 6. The current reference is set as 0.5 A with a hysteresis band of 10%. Switching is done at this band level. The torque waveform shows discontinuity due to small value of current reference.

**Figure 7:**
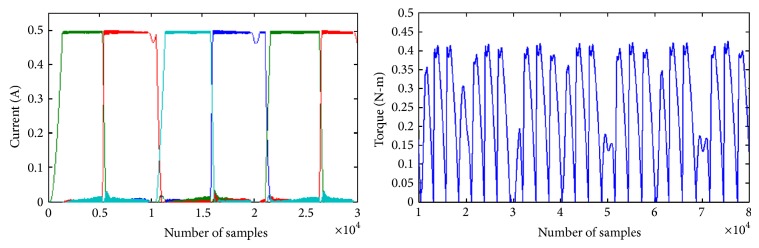
Phase currents and developed torque of SRM with 60 V sinusoidal excitation and constant current reference of 0.5 A.

Figure 7 shows the phase currents and developed torque waveforms of the modified R dump converter. The current waveform shows dips due to the magnitude variation in the supply voltage. The torque waveforms show less switching and the small peaks are at the zero crossing of the supply. Based on the current profile it may be seen that the DC excitation is having high torque ripple compared with sinusoidal excitation. The torque waveform shows a sharp peak due to which the ripple is higher. However in sinusoidal excitation the torque ripple is less. The smaller peaks are due to the zero crossing of the supply. But the average torque is high for AC excited SRM that can very well be seen in the tabulated values.

The values of average speed, speed ripple, average torque, and torque ripple are listed in Tables 2 and 3. It is found that the torque ripple is high at lower current reference and it can be reduced by keeping the current as high value. The speed ripple has also shown large value for smaller current reference. It is also observed that compared to DC excitation the sinusoidal excitation provides high average values for the torque and speed. In sinusoidal excitation ripples are also less significant when compared with the DC excitation. This is mainly due to the continuous excitation from the supply.

The SRM is tested for various current references, with different voltage magnitudes and the performance factors being tabulated. Table 4 shows the performance parameters of SRM which is excited with 100 V sinusoidal supply. As the reference current value is increased from 0.6 A to 1.5 A, it is observed that the speed ripple, torque ripple, and THD are reducing. At higher voltages the performance of the motor is improved. The power factor is also improved. The large harmonic distortion will lead to high losses and less efficiency. Due to the modified converter, current harmonics decrease with the increase in current reference which has reduced losses. Hence the efficiency of the drive is also improved.

By keeping a single DC current reference and varying the applied voltage the observed motor performance factors are tabulated in Table 5. It is found that the speed ripple, torque ripple, and THD are more as the voltage increased. The power factor is also poor. 

**Figure 8:**
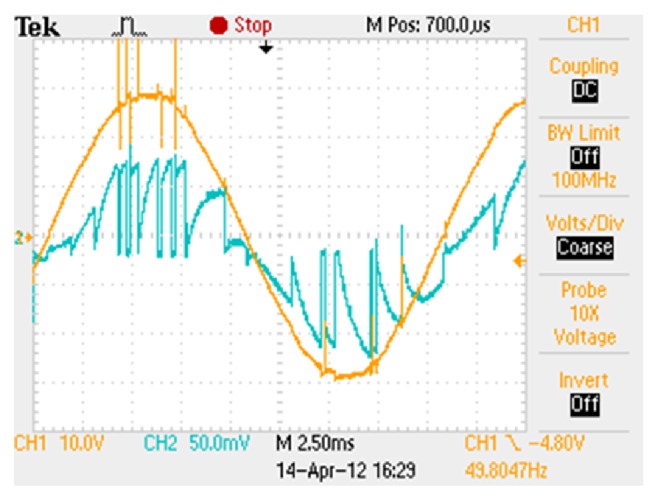
Supply voltage and current waveform of modified converter with sinusoidal current reference.

*Case (ii) DC and AC Excitation with Sinusoidal Current Reference*. With the same AC excitation the conventional and modified R dump converter configurations are chosen as given in Figures 1 and 2. The supply voltage and current waveforms for the modified converter configuration are shown in Figure 8. The sinusoidal current reference is fixed at a magnitude of 0.5 A.

**Figure 9:**
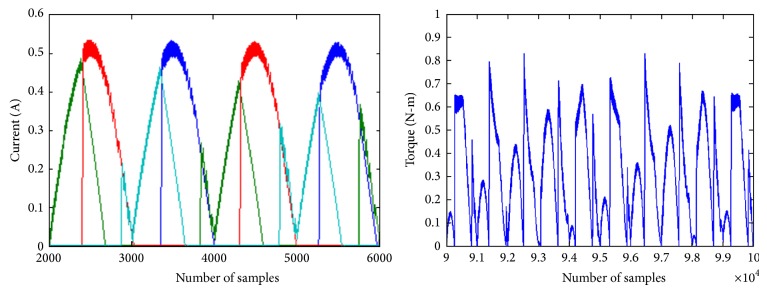
Phase currents and torque of SRM with 60 V DC excitation and sinusoidal current reference of 0.5 A.

**Figure 10:**
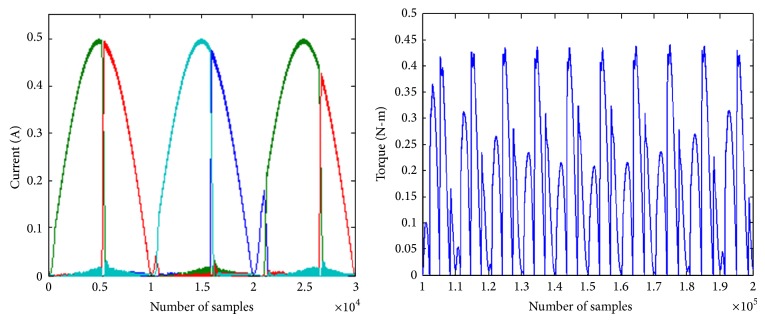
Phase currents and torque of SRM with 60 V sinusoidal excitation and sinusoidal current reference of 0.5 A.

To reduce iron losses it is preferred to have the currents be sinusoidal. Hence the analysis is repeated with sinusoidal current reference at both DC and sinusoidal excitation. The phase currents and developed torque of the SRM, under sine current reference with conventional and modified converter, are shown in Figures 9 and 10. Due to the current profile in DC excited SRM more torque ripples are observed.

Tables 6 and 7 present the average speed and average torque of the SRM when excited under DC and sinusoidal supply. It is found that the ripples in DC are high when compared with sinusoidal excitation. At high current reference the machine shows a good performance. Since the applied voltage magnitude is varying, the phase currents are also varying which provides more ripples in the torque profile. In spite of this, the required speed is achieved.

The machine performance based on power factor and THD is also obtained by exciting it with 100 V supply and various current references which are tabulated in Table 8. It is found that power factor is improved as the current reference is increased. It has also been observed that the current harmonics are reduced; hence the iron losses are reduced. The acoustic noise was also observed to be less in sinusoidal excitation which is not quantified.

**Figure 11:**
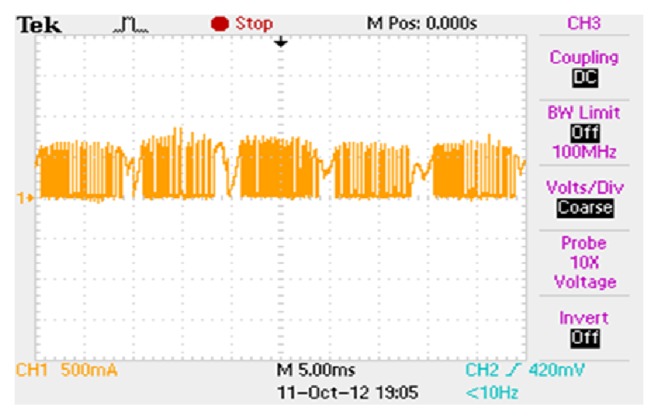
Phase currents for 0.5 A reference 60 V DC.

**Figure 12:**
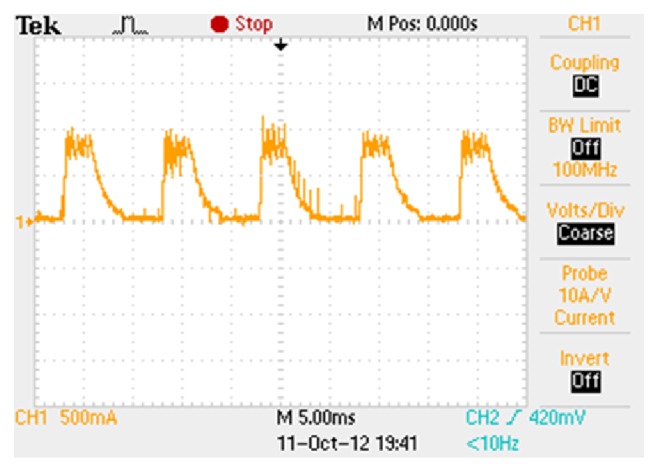
Phase B current waveform for 0.5 A reference 60 V DC.

The supply current is highly distorted due to the switching of the converter switches. Because of this the power factor is becoming poor and produces high harmonics. The supply current contains only third harmonics; hence lower iron loss is expected. The phase current waveform for a constant current reference of 0.5 A is shown in Figure 11. To have more understanding over the phase currents the phase B current alone is shown in Figure 12.

## 5. Experimental Setup

### 5.1. Design of Converter

The rating of transistor *T*_1_ is chosen to withstand the turnoff of transient voltage also. The current rating of the switch is chosen to withstand maximum phase current during turnon. The diodes of R dump are chosen to withstand freewheeling current. The dump resistor is chosen to limit the freewheeling current. The power dissipated on the dump resistor is high at lower speeds of the drive. Suitable circuits are presented in the literature to retrieve the power. The choice of power components is listed in Table 1.

### 5.2. System Configuration

Control part is organized through hardware in loop model. Commutations pulses are generated based on the information from position sensor which is acquired through a National Instrumentations Data Acquisition (NI DAQ) card PCI 6251. An R dump converter with necessary isolation and gate drivers are used to commutate the phase current. Hall effect current and voltage sensors are used for measuring the active phase voltage and current, respectively.

**Figure 13:**
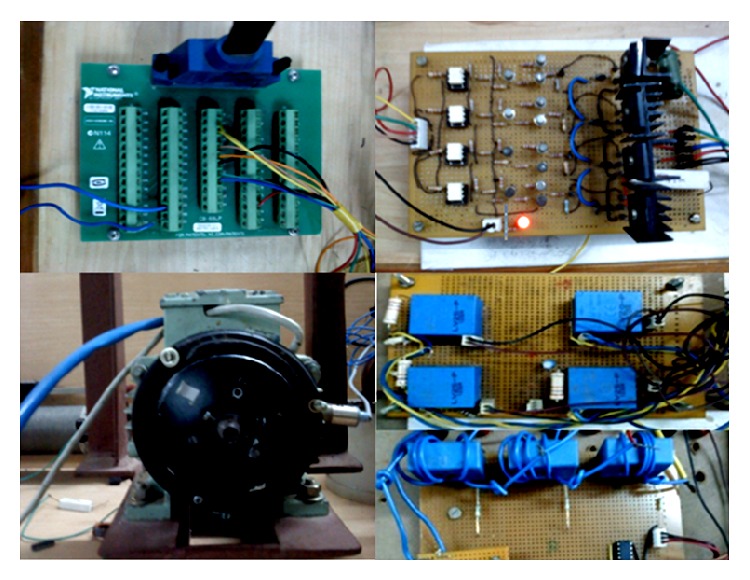
DAQ terminator, R dump converter, 8/6 SRM, and sensors.

**Figure 14:**
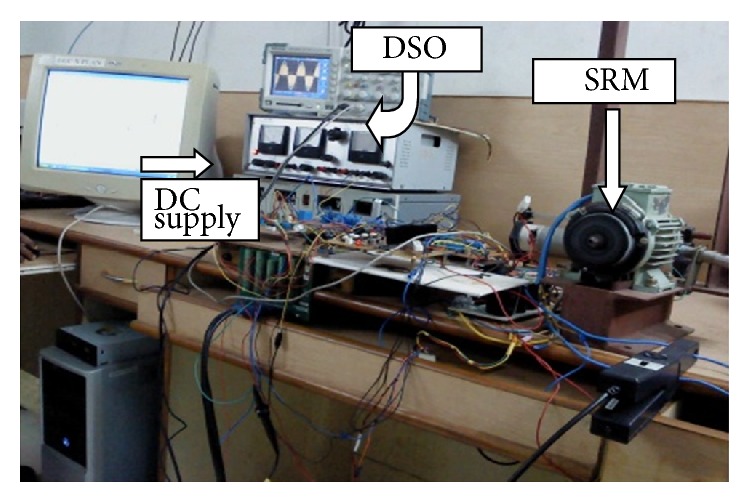
Hardware of modified R dump converter.

Hardware implementation of the modified R dump converter and its components are shown in Figures 13 and 14, respectively. The storage oscilloscope is used to capture the various waveforms. The current probe is used to measure the phase currents as well as the supply currents.

## 6. Conclusion

The electromagnetic performance of an 8/6 SRM with DC and sinusoidal excitation is investigated in terms of torque capability, torque ripple, average speed, and speed ripple. The standard single phase full bridge is employed to make unidirectional current flow in SRM phases. The DC link capacitance is removed which makes the converter weigh less and be cost effective. Lower torque and speed are observed in DC excitation. The iron loss with sinusoidal excitation is significantly reduced due to less current harmonics at high current reference. Thus the efficiency will be high at high speed. The modified converter can be used for fan type loads.

## Figures and Tables

**Table 1:** Switched reluctance motor, its hardware, and data acquisition specifications.

Motor specifications		Hardware configuration		Data acquisition specification	
Rated power	0.5 kW	Current sensor	LA-100P	DAQ board	NI PCI 6251
Rated current	3 A	Voltage sensor	LV25-P	Real-time interface	Embedded target
Stator poles	8	Optocoupler	MCT2E	Acquisition speed	100 *μ*s
Rotor poles	6	MOSFET	IRFP450		
Resistance/phase	2.67 Ω	Diodes	IN4007		
		Capacitor	1000 μF		

**Table 2:** Comparison of speed with DC and sinusoidal excitation with DC current reference.

Constant current reference (A)	Experimental analysis				Theoretical analysis			
	DC excitation (with capacitor)		Sinusoidal excitation (without capacitor)		DC excitation (with capacitor)		Sinusoidal excitation (without capacitor)	
	Average speed (RPM)	Speed ripple %	Average speed (RPM)	Speed ripple %	Average speed (RPM)	Speed ripple %	Average speed (RPM)	Speed ripple %
0.4	215	2.79	345	2.90	208	2.65	328	2.47
0.5	706	1.27	1829	1.20	659	1.17	1810	1.14
0.6	1518	0.46	2353	0.76	1478	0.41	2286	0.82
0.7	1973	0.35	2553	0.86	1952	0.32	2458	0.80
0.8	2307	1.52	2764	0.90	2295	1.57	2714	0.92
0.9	2469	0.81	2798	0.64	2423	0.88	2782	0.84
1.0	2510	0.84	2816	0.96	2498	0.85	2793	0.92
1.2	2620	0.88	2830	1.41	2601	0.83	2822	0.86
1.5	2934	1.29	2975	0.85	2896	0.81	2965	0.78

**Table 3:** Comparison of torque with DC and sinusoidal excitation with DC current reference.

Constant current reference (A)	Experimental analysis				Theoretical analysis			
	DC excitation (with capacitor)		Sinusoidal excitation (without capacitor)		DC excitation (with capacitor)		Sinusoidal excitation (without capacitor)	
	Torque average	Torque ripple %	Torque average	Torque ripple %	Torque average	Torque ripple %	Torque average	Torque ripple %
0.4	0.007	300.00	0.013	169.23	0.065	320.00	0.015	164.55
0.5	0.011	272.73	0.018	156.76	0.012	292.73	0.020	160.43
0.6	0.015	220.00	0.022	141.57	0.017	210.00	0.025	145.89
0.7	0.019	173.68	0.027	140.74	0.020	193.68	0.032	143.56
0.8	0.024	145.83	0.036	134.25	0.025	165.83	0.038	140.87
0.9	0.037	116.22	0.045	133.33	0.039	122.22	0.044	138.26
1.0	0.039	135.90	0.052	134.62	0.045	146.90	0.058	136.96
1.2	0.052	132.69	0.081	130.43	0.050	140.69	0.091	132.45
1.5	0.048	143.75	0.089	114.12	0.056	155.75	0.099	120.87

**Table 4:** 100 V sinusoidal supply with different DC current references.

Constant current reference (A)	Average speed (RPM)	Speed ripple %	Torque ripple %	Power factor	THD for current
0.6	1283	4.45	85.51	0.44	89.2
0.7	1847	1.18	69.69	0.52	84.7
0.8	2110	1.21	47.06	0.57	82.1
0.9	2343	2.28	37.45	0.62	79.6
1	2520	2.08	32.46	0.64	76.9
1.2	2747	0.96	22.18	0.7	71.6
1.5	2797	0.79	16.32	0.75	65.3

**Table 5:** 1 A constant current reference with different voltage magnitudes.

Applied voltage	Average speed (RPM)	Speed ripple %	Torque ripple %	Power factor	THD for current
30	1364	0.86	61.6	0.814	55.9
60	1768	0.96	134.6	0.746	66.7
75	2301	1.23	142.2	0.684	72.4
100	2587	1.68	156.4	0.648	75.9
125	2827	1.94	172.4	0.616	77.9
150	2961	2.42	184.6	0.587	80.7
175	3261	2.76	192.6	0.572	81.3
200	3547	3.13	204.6	0.564	81.7

**Table 6:** Comparison of speed with DC and sinusoidal excitation with sine current reference.

Sine current reference (A)	Experimental analysis				Theoretical analysis			
	DC excitation (with capacitor)		Sinusoidal excitation (without capacitor)		DC excitation (with capacitor)		Sinusoidal excitation (without capacitor)	
	Average speed (RPM)	Speed ripple %	Average speed (RPM)	Speed ripple %	Average speed (RPM)	Speed ripple %	Average speed (RPM)	Speed ripple %
0.4	315	6.79	445	4.90	332	6.43	462	5.60
0.5	816	4.27	1929	3.20	825	4.57	1935	3.70
0.6	1622	3.46	2453	1.76	1640	3.87	2420	1.86
0.7	2023	2.45	2753	0.86	2045	2.25	2732	0.76
0.8	2511	1.52	2864	0.90	2510	1.82	2837	0.80
0.9	2669	0.81	2898	0.64	2690	0.95	2878	0.74
1.0	2710	0.64	2916	0.66	2735	0.54	2903	0.65
1.2	2920	0.43	3254	0.41	2945	0.49	3276	0.48
1.5	3015	0.28	3685	0.25	3030	0.32	3665	0.29

**Table 7:** Comparison of torque with DC and sinusoidal excitation with sine current reference.

Constant current reference (A)	Experimental analysis				Theoretical analysis			
	DC excitation (with capacitor)		Sinusoidal excitation (without capacitor)		DC excitation (with capacitor)		Sinusoidal excitation (without capacitor)	
	Torque average	Torque ripple %	Torque average	Torque ripple %	Torque average	Torque ripple %	Torque average	Torque ripple %
0.4	0.01	320.00	0.018	189.23	0.013	302.00	0.016	189.23
0.5	0.016	292.73	0.022	176.76	0.015	272.73	0.024	176.76
0.6	0.018	245.00	0.025	161.57	0.019	255.00	0.026	161.57
0.7	0.021	203.68	0.027	150.74	0.023	223.28	0.028	150.74
0.8	0.026	165.83	0.039	144.25	0.029	155.38	0.043	144.25
0.9	0.037	136.22	0.045	133.33	0.040	127.73	0.049	133.33
1.0	0.041	125.90	0.057	122.62	0.046	112.45	0.061	122.62
1.2	0.046	102.69	0.085	100.43	0.049	99.86	0.082	100.43
1.5	0.048	83.75	0.089	88.12	0.052	76.88	0.093	88.12

**Table 8:** 100 V sinusoidal supply with various constant current references.

Sine current reference (A)	Average speed (RPM)	Speed ripple %	Torque ripple %	Power factor	THD for current %
0.6	2385	6.45	185.25	0.74	69.2
0.7	2567	4.19	169.69	0.78	64.7
0.8	2683	3.23	147.16	0.81	52.1
0.9	2891	2.78	137.25	0.83	48.6
1	3245	2.38	132.86	0.85	39.9
1.2	3675	1.96	122.28	0.87	35.6
1.5	3797	1.79	116.32	0.92	32.3
